# The Impact of Acute Postoperative Pain in Developing Chronic Pain after Total Knee Arthroplasty

**DOI:** 10.3390/neurolint16020034

**Published:** 2024-04-18

**Authors:** Nebojsa Nick Knezevic, Osman Syed, Christopher Kabir, Aisha Patel, Isabel Rao Shuai, Antony R. Tharian

**Affiliations:** 1 Department of Anesthesiology, Advocate Illinois Masonic Medical Center, Chicago, IL 60657, USA; osman.syed@midwestern.edu (O.S.); isabel.raoshuai@aah.org (I.R.S.); tonytharian@aol.com (A.R.T.); 2 Department of Surgery, University of Illinois, Chicago, IL 60612, USA; 3 Department of Anesthesiology, University of Illinois, Chicago, IL 60612, USA; 4 College of Osteopathic Medicine, Midwestern University, Downers Grove, IL 60515, USA; 5 Advocate Research Institute, Downers Grove, IL 60515, USA; christopher.kabir@aah.org

**Keywords:** CPSP, TKA, chronic pain, arthroplasty, postsurgical pain

## Abstract

While total knee arthroplasties (TKAs) are performed with the intent to reduce pain, chronic postsurgical pain (CPSP) is one of the most well-documented complications that can occur following surgery. This study aimed to assess whether perioperative factors, focusing on acute postsurgical pain and perioperative opioid consumption, were associated with the development of chronic postsurgical pain. Under general anesthesia, 108 patients underwent TKA and were treated postoperatively with a multimodal analgesia approach. Numeric Rating Scale (NRS) pain scores at rest and with movement were recorded on postoperative days 0–3, 7, 14, and 30. Patients were sent a survey to assess chronic pain at months 22–66, which was examined as a single-group post hoc analysis. Based on the responses, patients were either classified into the CPSP or non-CPSP patient group. Chronic postsurgical pain was defined as an NRS score ≥ 4 with movement and the presence of resting pain. The primary outcome was a change in NRS. There were no differences in NRS pain scores with movement in the first 30 days postoperatively between patients with CPSP and without CPSP. Each unit increase in resting pain on postoperative days 3 and 14 was associated with significantly greater odds of CPSP presence (OR = 1.52; OR = 1.61, respectively), with a trend towards greater odds of CPSP at days 7 and 30 (OR = 1.33; OR = 1.43, respectively). We found that very intense pain in the initial phase seems to be related to the development of CPSP after TKA.

## 1. Introduction

As the number of elective surgeries continues to increase yearly, more focus has turned towards monitoring complications and quality-of-life changes in postoperative patients. Chronic postsurgical pain (CPSP) is a frequently seen complication in patients who have undergone surgery. Only in 2019 was the definition of CPSP standardized, and it is now included in the 11th revision of the International Classification of Diseases. CPSP is defined as chronic pain that develops or increases in intensity after a surgical procedure or a tissue injury and persists beyond the healing process at least 3 months afterwards [1].

CPSP affects a substantial portion of patients and is a reported complication between 5% and 85% of the time, highly dependent on the type of operation [2,3]. Various studies have investigated pre- and postoperative risk factors associated with CPSP, including the severity of pre- and postoperative pain, preoperative functioning, age, gender, comorbidities, pain site distribution, depression, anxiety, number of pain sites, and pain [4,5,6,7,8,9,10]. The association between severe acute postoperative pain (APSP) and a higher likelihood of progression to CPSP has been observed across a range of surgeries, including hip arthroplasty, breast cancer surgeries, inguinal herniorrhaphy, thoracic surgeries, and cesarean sections [11].

The mechanism of CPSP is complex and most likely multifactorial. One theory is that high levels of perioperative pain can lead to chronic pain development because it not only limits rehabilitation but also causes central sensitization [12], a phenomenon where noxious stimuli change electrophysiology and neurotransmission in the brain, leading to hyperalgesia and enhanced temporal summation, thereby generating pain in the absence of inflammation [13]. This phenomenon is expected to develop after surgery and might not be evident initially. Consequentially, patients will present with a lower threshold for nerve activation, complaining of hyperalgesia allodynia and dysesthesia.

On a molecular level, the sensory perception of pain is carried by peripheral nociceptive fibers to the dorsal horn of the spinal cord. These afferent signals are transmitted to the spinal cord, up to the brainstem and cerebrum. These afferents will begin to fire rapidly during surgery in response to any damage to nerves [14]. Mice models have attempted to simulate the type of peripheral nerve insults that may be seen in a surgical setting, in order to monitor changes to the nerve. A mouse model study that preformed lesions to the sciatic nerve showed glutamate-induced neurodegenerative changes such as the loss of GABAergic interneurons. This interneuron loss in the superficial dorsal horn was found to be a promotional factor in the progression from acute to chronic neuropathic pain. The study also showed that mice with dysfunctional NMDA receptors (achieved via gene knockout) were protected from interneuron loss and, therefore, exhibited expected pain responses [15]. Unfortunately, clinical trials testing the role of perioperative medications to modulate NMDA receptors (either directly or via calcium channel modulation) have had limited proven efficacy. This may be due to a lack of quality studies, or because of the difficulty of standardizing surgeries and accounting for preoperative risk factors [16].

This paper specifically focuses on CPSP related to total knee arthroplasty (TKA) surgery. In the United States, total knee arthroplasty is one of the most performed orthopedic surgeries, with approximately 480,000 primary TKAs performed in 2019 and projections estimating between 800 thousand and 1.2 million TKAs annually in the 2040s/50s [17,18]. Studies have shown a high satisfaction rate in post-TKA patients (70–88%) based on pain reduction and functional improvement, and patients are often recorded as making notable progress in function and pain reduction between 3 to 6 months post-surgery [19,20,21,22,23,24,25]. Although most patients eventually endorse satisfaction with their TKA, undergoing the procedure has been shown to cause significant APSP [26]. Approximately 25% of TKA patients report persistent postsurgical pain, with 9–20% experiencing severe pain [27,28]. Significant predictors of CPSP when looking at TKA patients include moderate-to-severe APSP, younger age, and pain catastrophizing [29]. Therefore, effectively managing postoperative pain following TKA is crucial not only for ensuring patient satisfaction but also for facilitating recovery, as inadequate pain control may prolong hospitalization, delaying early ambulation and physical rehabilitation [30,31]. The consequences of prolonged hospitalization include increased costs and risks of complications associated with hospitalization—such as thromboembolic disease or infection [32].

There is also a monetary burden placed upon patients with CPSP. A prospective multicenter study investigating the direct and indirect costs of CPSP in a population of cardiac surgery patients found a mean monthly pain-related cost of CAD 207 in the first 6 months. Furthermore, the study reported that few patients utilized healthcare resources, and many were lost to follow-up. When considering the over USD half-billion costs associated with chronic pain across the USA, more research is needed in the less recognized CPSP subpopulation within the greater chronic pain group [33].

This study aimed to assess whether perioperative factors, focusing on acute postsurgical pain and perioperative opioid consumption, were associated with the development of chronic postsurgical pain. It was hypothesized that these pain scores would serve as predictors for the development of chronic pain.

## 2. Materials and Methods

This study conducted a retrospective cohort analysis utilizing prospectively collected data embedded within a larger RCT study design. The study was approved by the Advocate Health Care Institutional Review Board and informed consent was obtained from patients participating in the trial. Major inclusion criteria included age between 18 and 90 years and having undergone TKA. Exclusion criteria consisted of reoperation on the same knee, a body mass index (BMI) ≥ 45, radicular pain in the same leg, an allergy to local anesthetics, opioid habituation, pregnancy, the inability to communicate with hospital staff or investigators, and neuropathy of any etiology in the surgical extremity. Of the 120 patients recruited, 5 patients declined to participate and 7 did not meet the eligibility criteria. For the survey analysis, 103 patients were included who underwent total knee arthroplasty (TKA) and completed responses regarding their chronic postoperative pain.

Surgeries occurred at a large urban teaching hospital in Chicago, Illinois. The surgeries themselves were performed by a portion of the orthopedic surgery service at this center which was comprised of four physicians as well as their resident physicians.

Following surgery, pain assessments were conducted in the Post-Anesthesia Care Unit (PACU) upon arrival, and at 15, 30, 45, and 60 min, assessments were performed both at rest and during the movement of the affected joint. Pain medication use was also recorded. The Numeric Rating Scale (NRS) pain scale ranged from 0 to 10, with 0 indicating no pain, 1–3 indicating mild pain, 4–6 indicating moderate pain, 7–9 indicating severe pain, and 10 representing excruciating pain. Patients were treated with a multimodal regimen adhering to the enhanced recovery after surgery (ERAS) protocols. Supplemental pain medication was quantified in morphine equivalents. Patient satisfaction was evaluated on a scale of 1 to 5, with 5 indicating complete satisfaction, 4 satisfied, 3 somewhat satisfied, 2 dissatisfied, and 1 indicating complete dissatisfaction. Inpatient assessments were repeated at 12, 24, and 48 h post-surgery, reassessing the parameters mentioned above. The majority of patients had an inpatient admission length of approximately 48 h. Subsequently, patients were contacted via telephone for follow-up on postoperative days 3, 7, 14, and 30 after discharge from the hospital. In total, 103 patients out of 108 completed the 30-day follow-up after their discharge from the hospital.

In the second phase of data collection, patients were administered a modified Brief Pain Inventory questionnaire via mail, including queries on current medication usage [34], to assess postoperative pain and functioning, adhering to the applicable CONSORT guidelines.

The Brief Pain Inventory (BPI) is a questionnaire design originally designed for cancer patients by the WHO. The two categories of information collected in the BPI are pain intensity and pain interference. Intensity is measured in the range of 0–10, with 0 being “No Pain” and 10 being described as “Pain As Bad As You Can Imagine” and interference going from 0, “Does not interfere” to 10 “Completely Interferes”. Pain interference describes 7 different experiences in daily living: general activity, mood, walking ability, normal work, relations with other people, sleep, and the enjoyment of life [35].

The BPI, although originally designed for cancer patients, has been used across many specialties to assess pain across a variety of conditions. Multiple studies have shown its validity for a variety of conditions such as trials for low back pain [34], osteoarthritis [36], postoperative analgesia in CABG patients [37], and much more.

In cases where patients did not return the questionnaire by mail, a research team member initiated telephone follow-up. Patients were considered lost to follow-up after three unsuccessful attempts to contact them. Patients with completed surveys were categorized into two groups: those experiencing chronic pain and those without chronic pain.

This study uses a working definition of chronic postsurgical pain as a Numeric Rating Scale (NRS) score ≥ 4 with movement [27], with the presence of persistent resting pain. Furthermore, patients with pain that entirely resolved post-surgery but later recurred were excluded to account for the possibility of a new incident that aggravated a patient’s pain. This study also defined acute postsurgical pain as pain occurring within 30 days of surgery.

### Statistical Analysis

Descriptive statistics are presented as numbers and percentages for categorical variables, while means and standard deviations or medians and interquartile ranges were calculated for continuous data and NRS pain scores, depending on data normality.

Sample size calculations conducted post hoc for comparing CPSP and non-CPSP groups yielded an actual power of 0.97 for assessing a mean difference of 4 and 6, with a standard deviation of 2, an unequal group allocation ratio of 1:5, and a two-sided significance level of 0.05.

Sensitivity analysis for those lost to follow-up and differences in primary outcomes between CPSP groups were assessed using Student’s *t*-tests, the Wilcoxon Rank-Sum test, and the Chi-squared test of independence. Unadjusted (bivariate) logistic regression models were employed to examine the impact of clinical and patient factors such as age, BMI, and the quantity of opioid consumption on CPSP, with results reported as odds ratios with 95% confidence intervals. The threshold for statistical significance was set at a *p*-value < 0.05 and trend significance was set at a *p*-value < 0.1. Statistical analyses were conducted using SPSS 20 (IBM Corp., Armonk, NY, USA) and SAS 9.4 (SAS Institute, Cary, NC, USA).

## 3. Results

Of the 103 patients who completed their 30-day follow-up after TKA, 69 patients completed mail or telephone long-term follow-up. Long-term follow-up occurred between months 22 and 66 after total knee arthroplasty (TKA), dependent upon when subjects were enrolled. The analysis for variables of interest revealed no statistically significant differences in BMI, age, sex, or length of surgery between patients who completed follow-up and those lost to follow-up (Table 1).

Among the patients who completed long-term follow-up, the average age in years was 63.5 (1 standard deviation of 9.9 years). The population skewed female at 71% of respondents (49 patients). BMI was calculated at a median value of 32.9 kg/m^2^ (interquartile range of 9.4). The length of time required to complete the TKA was 103 min (interquartile range of 44 min).

According to our established definition, patients were categorized into two groups based on the presence or absence of chronic postsurgical pain (CPSP). Table 2 illustrates acute postsurgical Numeric Rating Scale (NRS) pain scores and postoperative opioid consumption between the CPSP and non-CPSP groups. No significant differences in morphine consumption were observed during the first three postoperative days. Notably, resting pain at days 3 and 14 emerged as predictive variables associated with CPSP occurrence, with higher resting pain ratings exhibiting a statistically significant association with an increased likelihood of chronic pain at day 3 (OR = 1.52; 95% CI = 1.02, 2.26) and day 14 (OR = 1.61; 95% CI = 1.10, 2.34). A non-statistically significant trend was identified between increasing pain scores and CPSP at days 7 and 30.

**Figure 1:**
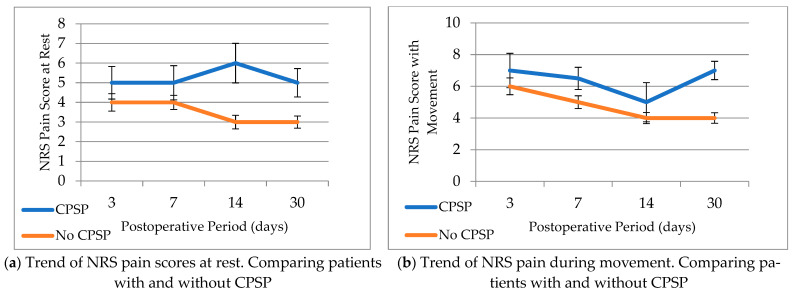
Changes over time for numeric pain rating scale at rest and in movement among patients with and without CPSP. Median scores with standard error at PODs 3, 7, 14, and 30 are reported (PODs 0–2 are not represented and no statistical difference was identified at these time points).

A difference was noted in median pain scores at rest between patients with CPSP and those without CPSP. These scores appeared to increase in patients with CPSP and stabilize or decrease in patients without CPSP (Figure 1). However, no significant differences were found in acute postoperative pain scores with movement.

Table 3 presents results from a subset of the Brief Pain Inventory and medication usage survey for 69 patients with CPSP, assessed at long-term follow-up. Among patients with CPSP, the interference of pain with activity, walking, normal work, and sleep was assessed. Pain had the highest interference with activity (7.46 out of 10), followed by normal work (6.77), walking (5.15), and sleep (3.83). Furthermore, 9 patients (13%) reported the use of opioid medications at this time.

## 4. Discussion

The incidence of CPSP in our final cohort of patients was 19%. This incidence is consistent with a multitude of studies and reviews. A large systematic review from 2023 involving 81 studies showed an incidence of 15.6% from a sample of 151,869 patients [38].

As previously mentioned, some risk factors for CPSP in the TKA population include younger age, pain catastrophizing, and moderate-to-severe APSP. Looking at APSP specifically, our study examines multiple follow-up time points between postoperative day (POD) 3 and the first month postoperatively following TKA. We found an association between APSP at rest on postoperative days 3, 7, 14, and 30 and the development of CPSP during our long-term follow-up period. There were OR = 1.52 and OR = 1.61 for developing CPSP for every one-point increase on the NRS pain scale at rest on POD 3 and POD 14. Generally, the pain scores between the two groups began to trend apart, with the CPSP group’s pain scores remaining high while the non-CPSP group’s trended downwards. A 2013 study using pain journals PODs 1–8 in TKA patients suggests this early deviation in pain trajectories may be a useful screening tool for the early detection of CPSP [39].

A prospective study by Buvanendran et al. included 245 patients undergoing primary TKA with the measurement of APSP as a weighted mean score in the first 72 h following surgery. They defined CPSP as an NRS pain with movement ≥ 4 at 6 months with a “consistent pattern of NRS pain at rest > 0 and at least one prior recoding of NRS pain with movement ≥ 4”. Using a binary logistic regression model that adjusted for numerous confounding factors, they reported an OR of 1.52 for the development of CPSP for every one-point increase in the measurement of APSP at rest. It was also noted that there were significant differences in patient NRS scores with CPSP versus non-CPSP at rest and in movement at the 3-week, 6-week, 3-month, and 6-month follow-up periods [40].

In a study primarily focused on the association between preoperative pain and the development of CPSP in TKA and total hip replacement (THR), Sayers et al. also collected data on APSP and found no association whatsoever between APSP and CPSP in TKA. In contrast to our data, they also reported that resting pain was associated with chronic pain following THR, and pain with movement was associated with chronic pain after TKA. They further discussed that osteoarthritis may have two pain mechanisms: subchondral bone pressure and inflammation, which could be more associated with resting versus moving pain [41].

These results highlight two points: the difference in associated pain while resting and moving, and the time frame in which follow-up is completed after a TKA.

The topic of pain at rest versus in movement as a risk factor for developing CPSP needs further research. Studies should consider the confounding variable of contralateral joint pain, as it is common practice to perform joint replacements one at a time, allowing for recovery before performing arthroplasty on the opposite joint. Future studies may also need to elucidate further what type of pain is occurring to better treat it, as many current studies group all pains, whether neuropathic, nociceptive, psychogenic, or otherwise, into the general categories of APSP and CPSP.

Our results also highlight that perioperative follow-up regarding pain alleviation is essential. In the aforementioned Sayers et al. paper, the authors made the significant conclusion that preoperative pain upon movement was their strongest predictor of CPSP when specifically looking at TKA [41]. Furthermore, in a retrospective cohort study of 1504 patients, Pua and Ong stressed the importance of early mobilization after TKA. The study showed that as little as a 1-day difference (postoperative day 1 versus postoperative day 2) in the initiation of mobilization was associated with a shorter length of stay, lower costs, and improved knee function (increased odds of achieving at least 90 degrees of knee flexion by 33%) [42]. APSP is likely an impeding factor to continue with expected rehabilitation and mobilization. As the Pua paper shows the importance of ambulation and the Sayers paper shows the importance of preoperative pain on ambulation, we believe that pain control is paramount during this perioperative period. Pain control can allow patients to ambulate earlier, participate in rehab, and hopefully have a lower likelihood of developing CPSP.

To our knowledge, there are no current society practice guidelines detailing a schedule for continuous short-term follow-up after TKA to address pain symptoms directly. Much of the literature, including a recent review article regarding follow-up, is centered primarily on surveillance to prevent long-term complications that could lead to a need for revision surgery [43].

An early postoperative clinic follow-up may be warranted, focusing on wound healing and pain management. The Toronto General Hospital opened a first-of-its-kind Transitional Pain Service in 2014. Under their model, patients receive coordinated interdisciplinary care pre- and postoperatively for patients at increased risk. They also manage opioids for complex patients and “improve patient coping and functioning” [44]. The data from their first two trials show promising outcomes for their patients. The first trial focused on the role of concomitant psychological services during transitional clinic visits. Patients who opted to receive psychological services, when compared to patients who did not, showed greater reductions in opioid use and pain interference. They also had the added benefit of a significant reduction in depressed mood before transitioning from the clinic [45].

A second study, published in 2018, compared opioid weaning rates 6 months after surgery in transitional pain service patients who were opioid-naive versus experienced. Opioid-naive patients reduced consumption by 69% compared to 44% in the opioid-experienced population; 46% and 26%, respectively, weaned completely. These studies, although from a single center, are promising indicators that the increased perioperative management and surveillance of surgical patients can lead to improved outcomes [46].

It is important to ask who manages sub-acute postoperative pain: an anesthesiologist or the operating surgeon. A follow-up appointment for this sub-acute time frame may be established early within the post-op period to monitor pain management closely. These clinics could be an important implementation to bring into practice to address a crucial time frame to control pain and reduce the risk of developing CPSP. At the time of writing, there do not seem to be universal follow-up guidelines for orthopedic surgeons following TKA; the American Association of Hip and Knee Surgeons recommends a two- to three-week follow-up for wound checks, but notes this as optional [47,48]. Furthermore, the Centers for Medicare and Medicaid Services currently incentivizes early visits after leaving the inpatient setting but still only requires a face-to-face visit within two weeks of discharge. Patients may not necessarily require a face-to-face visit, but the early postoperative period is clearly critical, and some early intervention is important to the prevention of CPSP.

A study by Fransen et al. that was focused on fast-track protocols for total knee arthroplasty involved interoperative regional anesthesia, a multimodal pain regimen with as-needed pain medication (versus scheduled opioids), and earlier joint loading exercise, with a 2-week outpatient follow-up. The study participants also maintained contact with the surgeon’s clinic and kept a pain diary. The study showed significantly lower VAS scores for knee pain, as well as more functional mobility in the first week as compared to standard protocol patients [49]. This may be an excellent alternative for surgeons who desire to maintain the pain management of their own patients but also want to implement changes to their practice to best prevent the development of CPSP in their TKA patients.

As most studies focus on reactionary treatments in the postoperative period, it would make sense to consider prophylactic measures against CPSP. Unfortunately, the current data are mostly of little clinical significance. A 2021 systematic review and meta-analysis of 110 double-blind, placebo-controlled RCTs evaluating perioperative systemic drugs concluded, “Based on currently available evidence, none of the drugs studied so far can be recommended for clinical use specifically for the indication of preventing chronic pain after surgery” [16]. Similarly, a Cochrane review looking at regional anesthesia, rather than medication-based therapy, found some evidence for regional blocks in thoracotomy and breast cancer surgery but stated their conclusions to be fairly weak due to a general paucity of quality trials [50].

The results of our study are limited by the lack of adjustment for confounding variables such as other previously studied risk factors for CPSP following TKA. Other limitations of this study include the modest sample size of patients at our long-term follow-up period due to the 32% of patients that were lost to follow-up, missing data from our long-term follow-up period, and the lack of data on morphine equivalents concerning opioid consumption in our long-term follow-up period. One significant limitation of our study is its need for internal validity. The pain scores at rest leading to CSPS were statistically significant for days 3 and 14; however, the pain scores during movement that influenced the development of CSPS were statistically significant for day 30. The lack of internal validity could be addressed by performing a study with more data. Another limitation is that it is a retrospective study that can only establish association and not causality. Finally, the large ratio of time for long-term follow-up (22–66 months) means that patients may have had different results if the time until each response was standardized across the cohort.

## 5. Conclusions

We found that very intense pain in the initial phase seems to be related to the development of CPSP after TKA. Physicians may consider closely monitoring patients during this time for pain management with particular attention to changes in resting acute pain. Further investigations are needed to determine if improving analgesia during acute postoperative time with various blocking techniques can prevent the development of chronic pain. To promote the continuity of care, additional acute and chronic care studies are needed to inform care practices at transitional pain clinics.

## Figures and Tables

**Table 1:** Sensitivity analysis assessing patient characteristics, length of surgery, and pain scores of subjects with and without long-term follow-up.

Characteristics	LTFU (n = 69)	No LTFU (n = 34)	*p* Value
Age (mean years ± SD)	63.5 ± 9.9	63.0 ± 11.6	0.829
Sex n (%)			0.082
Male	20 (29%)	16 (47%)	
Female	49 (71%)	18 (53%)	
BMI kg/m^2^, median (IQR)	32.9 (9.4)	34.0 (6.4)	0.672
Length of surgery min, median (IQR)	103.0 (44)	96.0 (29)	1.000
NRS Pain Score at Rest, median (IQR)			
POD 0	1.0 (6)	1.0 (5)	0.864
POD 1	5.0 (3)	4.0 (3)	0.837
POD 2	4.0 (4)	4.0 (4)	0.935
POD 3	4.5 (2)	5.0 (5)	0.269
POD 7	4.0 (3)	5.0 (5)	0.187
POD 14	3.0 (3)	3.0 (4)	0.880
POD 30	3.0 (4)	3.0 (4)	0.958
NRS Pain Score with Movement, median (IQR)			
POD 0	1.0 (5)	1.5 (6)	0.479
POD 1	6.0 (3)	5.0 (3)	0.958
POD 2	6.0 (3)	4.0 (4)	0.607
POD 3	6.0 (3)	6.0 (4)	0.856
POD 7	6.0 (3)	6.0 (5)	0.422
POD 14	4.0 (3)	4.0 (3)	0.880
POD 30	4.0 (5)	4.0 (3)	0.788

IQR = Interquartile Range, LTFU = Long-term Follow-up, POD = Postoperative Day, SD = Standard Deviation.

**Table 2:** Bivariate logistic regression model for variables of interest and pain scores on chronic postsurgical pain.

Characteristics	OR (95% CI)
Age (years)	1.03 (0.97, 1.10)
Female sex	2.68 (0.54, 13.37)
BMI (kg/m^2^)	1.03 (0.96, 1.11)
Morphine equivalents	
POD 0	1.010 (0.99, 1.03)
POD 1	0.997 (0.97, 1.02)
POD 2	0.997 (0.97, 1.02)
POD 3	0.957 (0.89, 1.03)
NRS Pain Score at Rest	
POD 0	0.99 (0.84, 1.16)
POD 1	0.99 (0.84, 1.16)
POD 2	1.11 (0.80, 1.53)
POD 3	1.52 (1.02, 2.26) **
POD 7	1.33 (0.98, 1.79) *
POD 14	1.61 (1.10, 2.34) **
POD 30	1.43 (0.99, 2.07) *
NRS Movement Pain Score	
POD 0	1.02 (0.82, 1.27)
POD 1	1.26 (0.88,1.79)
POD 2	1.07 (0.77, 1.48)
POD 3	1.14 (0.81, 1.62)
POD 7	1.14 (0.85, 1.53)
POD 14	1.18 (0.89, 1.57)
POD 30	1.51 (1.02, 2.25)

Abbreviations: OR = Odds Ratio, BMI = Body Mass Index, POD = Postoperative Day, NRS = Numeric Response * *p* < 0.1, ** *p* < 0.05.

**Table 3:** Brief Pain Inventory score and medication usage of subjects with CPSP.

Interference of Activity (mean ± SD)	7.46 ± 1.74
Interference of Walking (mean ± SD)	5.15 ± 3.08
Interference of Normal Work (mean ± SD)	6.77 ± 2.62
Interference of Sleep (mean ± SD)	3.83 ± 2.64
Medication Use n (%)	
Opioids	9 (13.0%)
Non-opioids	4 (5.8%)

CPSP = Chronic Postsurgical Pain, SD = Standard Deviation.

## Data Availability

The datasets generated during and/or analyzed during the current study are available from the corresponding author on reasonable request.
